# Zimbabwe adverse events following immunisation surveillance system: A descriptive study with COVID-19 vaccine safety updates

**DOI:** 10.4102/sajid.v41i1.785

**Published:** 2026-03-20

**Authors:** Priscilla P.M. Nyambayo, Rumbidzai Manyevere, Libert Chirinda, Steny F. Marekera, Tatenda Nyamandi, Rutendo P. Chaitezvi, Richard T. Rukwata, Ushma Mehta, Michael S. Gold

**Affiliations:** 1 Medicines Control Authority of Zimbabwe, Harare, Zimbabwe; 2 Centre for Infectious Disease Epidemiology and Research, School of Public Health and Family Medicine, University of Cape Town, Cape Town, South Africa; 3 Adelaide Medical School, Faculty of Health and Medical Sciences, The University of Adelaide, Adelaide, Australia

**Keywords:** AEFI surveillance system, AEFI causality assessment, mHealth active participant-centred (MAPC) AEFI surveillance, VigiGrade^®^ completeness score and WHO Global Benchmarking Tool (GBT) Version VI

## Abstract

**Background:**

A functional national adverse events following immunisation (AEFI) surveillance system is vital for guiding vaccination policies and sustaining public confidence. The system helps ensure safe immunisation delivery, thereby maintaining high delivery vaccine coverage and reducing vaccine-preventable diseases (VPDs). Optimising these systems remains a critical public health priority.

**Objectives:**

This novel study reviews and evaluates Zimbabwe’s AEFI surveillance system from 1998–2024, including updates on coronavirus disease 2019 (COVID-19) vaccine safety to identify strengths, weaknesses and opportunities for improvement.

**Method:**

We conducted an in-depth analysis of all AEFI reports received from 1998–2024, assessing reporting trends, overall system performance AEFI investigation, causality assessment and feedback to reporters using the World Health Organization (WHO) Global Benchmarking Tool (GBT). Duplications were excluded, and reports with evidence of AEFI(s) after vaccination were included.

**Results:**

The findings show a steady increase in AEFI reports per annum, especially from 2006–2024, with substantial increases in 2023 and 2024, reaching rates of 64 and 82 reports per 100 000 surviving infants. The reporting rate exceeded the WHO-recommended minimum AEFI reporting rate in 13 years (50%) out of the 26 years. The COVID-19 vaccination programme generated 519 AEFI reports (23%) between 2021–2023, with reporting rates of 2.1, 0.1 and 1.1 per 100 000 population, respectively.

**Conclusion:**

A strong partnership between the immunisation programme and regulatory authority has enhanced AEFI surveillance. However, incomplete AEFI case investigations and delays in AEFIs detection remain key. System improvements should incorporate digital tools to enhance reporting investigation and signal detection, including postmortem examinations for serious AEFIs.

**Contribution:**

The unique Zimbabwe AEFI publication contributes to the scientific knowledge, challenges and potential signals to heed to enhancing vaccine safety systems.

## Introduction

Globally, immunisation is one of the most cost-effective ways of preventing or reducing the severity of infectious diseases. Ensuring that vaccines are safe and effective is a responsibility shared by manufacturers, national immunisation programmes (NIPs) and the national medicines regulatory agencies (NMRAs).^1^ Timely detection and investigation of adverse events following immunisation (AEFIs), causality assessment, identification of signals, response and appropriate communication are essential components of a robust surveillance system.^2,3^ Not uncommonly, AEFIs result in diminished public trust and negatively impact vaccine coverage.^4,5,6^

**FIGURE 1:**
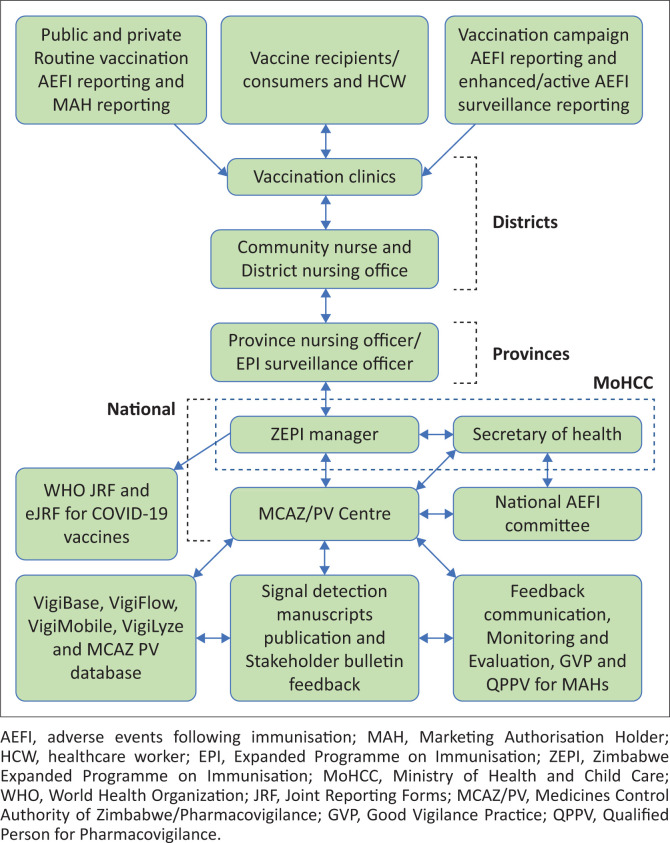
Zimbabwe adverse events following immunisation surveillance process flow from vaccination sites to Zimbabwe Expanded Programme on Immunisation and Medicines Control Authority of Zimbabwe National PV Centre.

The African region contributes a cumulative total of only 0.9% individual case safety reports (ICSRs) to VigiBase^®^, the World Health Organization (WHO) global surveillance safety database.^7^ Most of these reports relate to medicines rather than vaccines.^7^ Robust evidence on the performance of vaccine safety surveillance is lacking in most low- and middle-income countries (LMICs). In Zimbabwe, AEFI surveillance is overseen by the NMRA, which is the Medicines Control Authority of Zimbabwe (MCAZ), in partnership with the Zimbabwe Expanded Programme on Immunisation (ZEPI), within the Ministry of Health and Child Care (MoHCC).^8,9,10^ The MCAZ National Pharmacovigilance Centre (NPC) was delegated the responsibility of overseeing AEFI surveillance since 1998 and is a full member of the WHO International Drug Monitoring Program.^7,11,12^ MCAZ processes all AEFIs received from ZEPI for causality assessment by the National AEFI Committee, which reviews cases monthly or ad hoc if deemed necessary for fatal cases or those causing community concern (Figure 1).

As a contributing member of the global database, Zimbabwe transmits all ICSRs electronically to VigiBase^®^ and aggregated electronic Joint Reporting Forms (eJRF) and electronic AEFI data to the WHO Headquarters (HQ) vaccine safety database. These data are used for the global indicators of vaccine safety surveillance and to assess trends in AEFI reporting for signal detection.^7,10,12,13^

The aim of our study was to provide a descriptive review of the Zimbabwean AEFI surveillance system from 1998 to 2024, highlighting the strengths, weakness and opportunities for improvement. The specific aims were to describe the following:

- The AEFI reports received according to the demographic characteristics of the vaccinees, the geographical distribution of reporting, suspected vaccine(s) and types of AEFIs reported according to the Medical Dictionary for Regulatory Activities (MedDRA) system organ classification (SOC) and Preferred Terms (PTs).
- The trends in the national AEFI reporting rate per 100 000 surviving infants between 1998 and December 2024 for childhood vaccines and for adults after COVID-19 vaccines using the vaccinated population.
- The AEFI causality assessment system, providing a synopsis of cause-specific AEFI classifications, including immunisation errors and clusters identified through spontaneous reporting.
- The quality of the Zimbabwean AEFI reports as determined by the VigiGrade^®^ completeness score in the VigiBase^®^ database.
- The performance of the AEFI system in relation to, and as assessed by, the WHO Global Benchmarking Tool (GBT) AEFI surveillance indicators.

## Research methods and design

Approximately, 300 000–550 000 Zimbabwean children below 18 years are vaccinated annually, with vaccine coverage rates of 80% – 95% per vaccine, according to the Global Alliance for Vaccines and Immunisation (GAVI) annual reports from 2012 to December 2024. In addition, 6 429 117 adults received first doses of COVID-19 vaccines and 4 785 620 received second doses from February 2021 to December 2023. All deduplicated AEFI reports received by the MCAZ NPC and ZEPI from 1998 to December 2024 were uploaded to the in-house e-pharmacovigilance (e-PV) system. The AEFI reports were analysed based on their seriousness, type of vaccine and type of AEFI reported and were classified according to the MedDRA SOC and PTs and demographic data of the vaccines.^14,15^ The annual AEFI reporting rates were calculated separately for childhood and COVID-19 vaccines. The AEFI reporting rate for childhood vaccines was calculated by dividing the total number of paediatric AEFI (serious and non-serious) reports received in a year by the total number of surviving infants per year and reported as per 100 000 surviving infants per year (based on annual UNDP statistics for surviving infants in Zimbabwe from 1998 to 2021). The AEFI reporting rate for COVID-19 vaccines, administered to adults, was calculated by dividing the total number of COVID-19 AEFI reports received by the total number of any COVID-19 vaccine doses administered to adults.^16,17^ Causality assessment was done by the trained National AEFI Committee using the WHO AEFI causality assessment methodology (2019) and protocols.^18,19,20^

We assessed the ability of the system to detect immunisation errors and clusters as identified by the AEFI National Committee. The assessment of completeness and quality of the AEFIs reports was determined by the VigiBase^®^ VigiGrade completeness score.^21^ The maximum VigiGrade completeness score is 1, and the minimum is zero based on four AEFI completeness criteria: patient information (sex, age, medical history, concurrent conditions); adverse event information (event description, outcome of reaction); medicine and vaccine information (vaccine generic or trade name, time to onset, indication for use) and availability of additional information (challenge, rechallenge, case narrative, AEFI case investigation, laboratory results, including postmortem reports).^21^ The quality of an AEFI report determines the extent to which the report can be reliably assessed for causality and incorporated into risk–benefit decision-making.^21^ The annual median score of the VigiGrade completeness was measured for AEFIs only for the purpose of this report.

The geographical distribution and frequency of AEFI reports based on the reporting facility and province(s) from which these reports arose were reflected in a heat map.

The WHO GBT is an objective tool for evaluating national regulatory systems, identifying strengths and opportunities and building regulatory capacity for medicines and vaccines, including AEFI surveillance, harmonisation and reliance.^22^ We used the results of the independent WHO GBT assessment of MCAZ’s NPC in December 2024 to further reflect on the performance of the AEFI system. Finally, we identified opportunities to strengthen the national AEFI system by examining gaps and weaknesses identified in the AEFI system, including those highlighted by the WHO GBT vigilance indicators.

### Ethical considerations

Ethical clearance to conduct this study was obtained from the Medical Research Council of Zimbabwe (reference number: MRCZ/A/2268) and the University of Cape Town Human Research Ethics Committee (reference number: HREC 184/2020).

## Results

**FIGURE 2:**
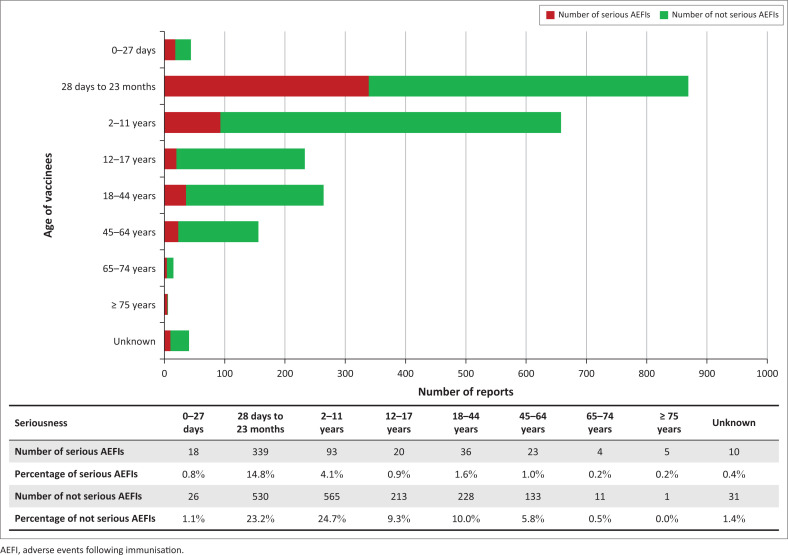
Age of vaccinees and severity of adverse events following immunisations.

From September 1998 to December 2024, 8181 ICSRs were received by the MCAZ NPC, of which 2286 (30.0%) were AEFIs, with 548 of these classified as serious. No pregnancy-associated AEFIs were reported. The demographic characteristics of the vaccinees were as follows: 1052 (46%) were male, 1180 (52%) were female and 54 (2%) were of unknown gender. Most AEFI reports (*n* = 1767; 77%) occurred after routine childhood vaccination in infants between 28 days and ≤ 24 months (38%) and children from ≥ 2 to ≤ 11 years (29%). Adult reports (18–44 years) accounted for 12% of all reports (Figure 2).

**FIGURE 3:**
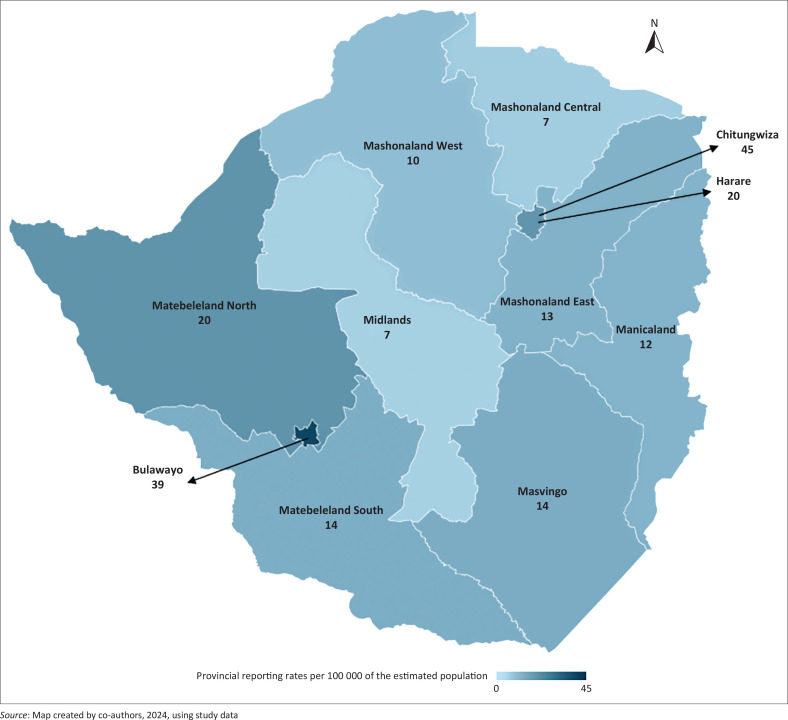
Geographical distribution – Zimbabwe provincial adverse events following immunisation reporting rates per 100 000 estimated population from 1998 to 2024.

Adverse events following immunisation reports were received from all 11 provinces in Zimbabwe, with 435 reports (19%) from the capital, Harare. Only 22 (1%) of the AEFI reports were not identified by location (Figure 3).

Oral polio vaccines were the most frequently reported vaccine associated with AEFIs (*n* = 647; 31.2% of all AEFI reports) (Table 1). Coronavirus disease 2019 vaccines were the second most reported vaccine (*n* = 430; 20.7% of all AEFI reports), followed by the measles and rubella vaccine (15.1%), the pentavalent (diphtheria, tetanus, pertussis, hepatitis B, *Haemophilus influenzae* B) vaccine (14.7%), measles vaccine (11%), pneumococcal vaccine (9.3%), typhoid vaccine (6.1%), diptheria, tetanus and pertussis (DTP) vaccine (5.6%), rotavirus vaccine (5.1%), *Bacillus* Calmette-Guérin (BCG) vaccine (2.6%) and human papillomavirus (HPV) vaccine (2.3%).

**TABLE 1:** Adverse events following immunisation reports of suspected vaccines and co-reported vaccines.

Co-reported active ingredients (WHODrug)	Suspected or interacting vaccine (*n*)	Co-reported active vaccines (*n*)	Total	% Suspected/interacting vaccine	% Co-reported medicines or vaccines
Polio vaccine	647	47	694	31.2	2.3
COVID-19 vaccine	430	0	430	20.7	0.0
Measles vaccine; Rubella vaccine	312	2	314	15.1	0.1
Diphtheria vaccine; Hepatitis B vaccine; Hib vaccine; Pertussis vaccine; Tetanus vaccine	305	1	306	14.7	0.0
Measles vaccine	228	1	229	11.0	0.0
Pneumococcal vaccine	192	13	205	9.3	0.6
Typhoid vaccine	127	1	128	6.1	0.0
Diphtheria vaccine; Pertussis vaccine; Tetanus vaccine	116	1	117	5.6	0.0
Rotavirus vaccine	106	20	125	5.1	1.0
BCG vaccine	53	1	54	2.6	0.0
HPV vaccine	48	0	48	2.3	0.0
Cholera vaccine	41	0	41	2.0	0.0
Bacterial and viral vaccines, combined	35	1	36	1.7	0.0
Tetanus vaccine	20	0	20	1.0	0.0
Hepatitis B vaccine	18	0	18	0.9	0.0
Diphtheria vaccine; Tetanus vaccine	15	1	16	0.7	0.0
Diphtheria vaccine	11	0	11	0.5	0.0
Diphtheria vaccine; Hib vaccine; Pertussis vaccine; Tetanus vaccine	9	0	9	0.4	0.0
Diphtheria vaccine; Hepatitis B vaccine; Pertussis vaccine; Tetanus vaccine	6	0	6	0.3	0.0
Measles vaccine; Mumps vaccine; Rubella vaccine	3	0	3	0.1	0.0
Rabies vaccine	3	0	3	0.1	0.0
Diphtheria vaccine; Hib vaccine; Pertussis vaccine; Polio vaccine; Tetanus vaccine	2	0	2	0.1	0.0
Hepatitis A vaccine	1	0	1	0.0	0.0
Hib vaccine	1	0	1	0.0	0.0
Measles vaccine; Mumps vaccine; Rubella vaccine; Varicella zoster vaccine	1	0	1	0.0	0.0
Rubella vaccine	1	0	1	0.0	0.0

BCG, Bacillus Calmette-Guérin; HPV, human papillomavirus; Hib, Haemophilus influenzae type b.

Most AEFIs, as classified by MedDRA SOC, included general disorders and administration site reactions (31.5%) encompassing events such as local injection site reactions (pain, swelling and reduced mobility) and persistent crying (Table 2).

**TABLE 2:** Adverse events following immunisation reports received between 1998 and 2024 distributed by MedDRA system organ classifications.

Reaction (MedDRA)	Reports	
	*n*	%
General disorders and administration site conditions	653	31.5
Skin and subcutaneous tissue disorders	458	22.1
Gastrointestinal disorders	357	17.2
Nervous system disorders	252	12.2
Infections and infestations	240	11.6
Respiratory, thoracic and mediastinal disorders	113	5.5
Eye disorders	46	2.2
Metabolism and nutrition disorders	44	2.1
Musculoskeletal and connective tissue disorders	37	1.8
Immune system disorders	14	0.7
Investigations	11	0.5
Vascular disorders	10	0.5
Cardiac disorders	8	0.4
Psychiatric disorders	8	0.4
Blood and lymphatic system disorders	6	0.3
Reproductive system and breast disorders	5	0.2
Renal and urinary disorders	4	0.2
Hepatobiliary disorders	3	0.1
Congenital, familial and genetic disorders	2	0.1
Ear and labyrinth disorders	2	0.1
Injury, poisoning and procedural complications	1	0.0
Neoplasms benign, malignant and unspecified (incl cysts and polyps)	1	0.0

MedDRA, Medical Dictionary for Regulatory Activities.

**FIGURE 4:**
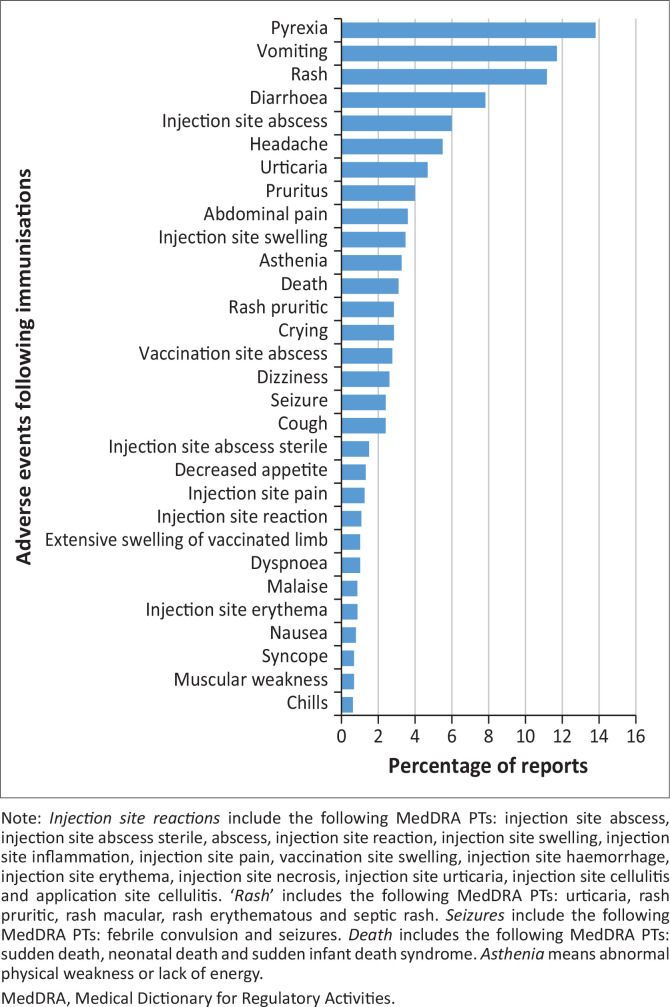
Frequency of adverse events following immunisations types reported according to MedDRA Preferred Terms.

Additional categories included skin and subcutaneous tissue disorders (22.1%), gastrointestinal disorders (17.2%), nervous system disorders (12.2%) and infections or infestations (11.6%). The reported frequency of suspected AEFIs grouped according to MedDRA PTs included pyrexia (13.8%), vomiting (11.7%), rash (11.2%), diarrhoea (7.8%), injection site abscess (6.0%), headache (5.5%), urticaria (4.7%) and pruritus (4.0%) (Figure 4).

**FIGURE 5:**
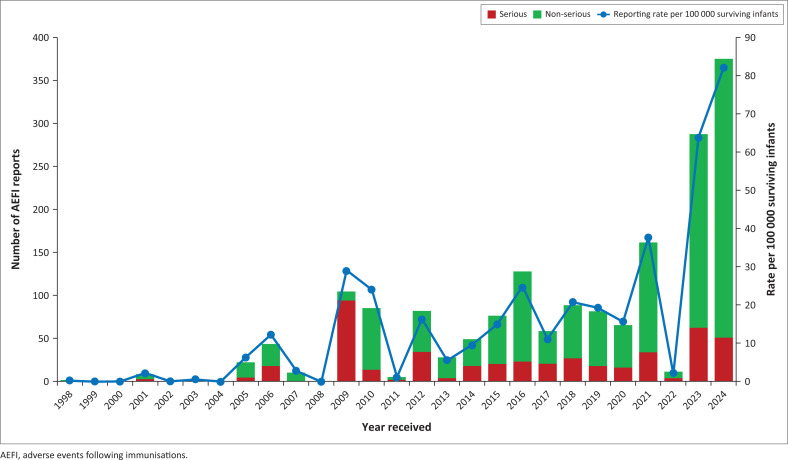
Annual adverse events following immunisation (excluding COVID-19 vaccines) reporting rates, serious and non-serious adverse events following immunisations from 1998 to 2024.

The annual AEFI reporting rates ranged from 0 to 82 per 100 000 surviving infants per year, with peak reporting rates noted in 2009 (*n* = 29), 2010 (*n* = 24), 2016 (*n* = 25), 2021 (*n* = 37), 2023 (*n* = 64) and the highest in 2024 (*n* = 82) (Figure 5).

**FIGURE 6:**
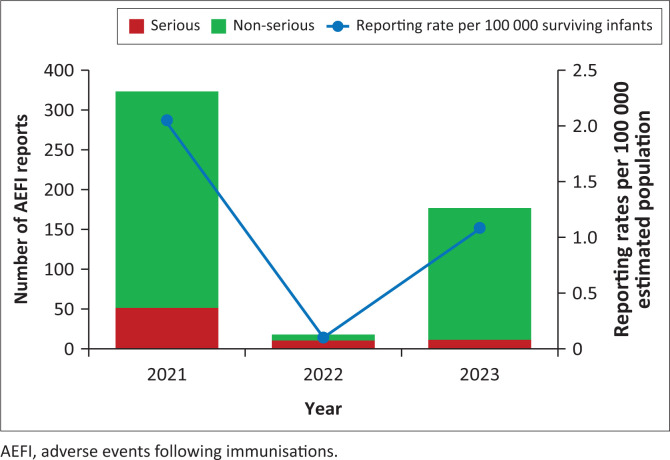
Annual COVID-19 adverse events following immunisation reporting rates, serious and non-serious adverse events following immunisations from 1998 to 2023.

The reporting rates exceeded the WHO-recommended minimum AEFI reporting rate of 10 per 100 000 surviving infants for 13 years of the 26 years (50%) since beginning the surveillance system in 1998. The adult COVID-19 vaccination programme yielded 519 reports (23% of all AEFI reports) over the 3-year period from 2021 to 2023, with AEFI reporting rates of 2.1 (in 2021), 0.1 (in 2022) and 1.1 (in 2023) per 100 000 estimated population (Figure 6). Rates for children’s vaccines were calculated using the number of surviving infants. Rates for COVID-19 vaccines were calculated using the estimated population.

In terms of seriousness, 1738 (76%) AEFI reports were non-serious, and 548 (24%) were serious. Of the serious reports, 174 out of 548 (32%) were registered fatalities and 99 out of 548 (18%) registered prolonged hospitalisation (Table 3).

**TABLE 3:** Categories of seriousness of suspected adverse events following immunisations received by Medicines Control Authority of Zimbabwe National Pharmacovigilance Centre from 1998 to 2024.

Seriousness criteria	Serious AEFI reports	
	*n*	%
Death	174	32
Life threatening	8	1
Caused, prolonged hospitalisation	99	18
Disabling, incapacitating	12	2
Congenital anomaly, birthdefect	0	0
Other medically important condition	255	47

**Total serious AEFIs**	**548**	**100**

AEFI, adverse events following immunisation.

**FIGURE 7:**
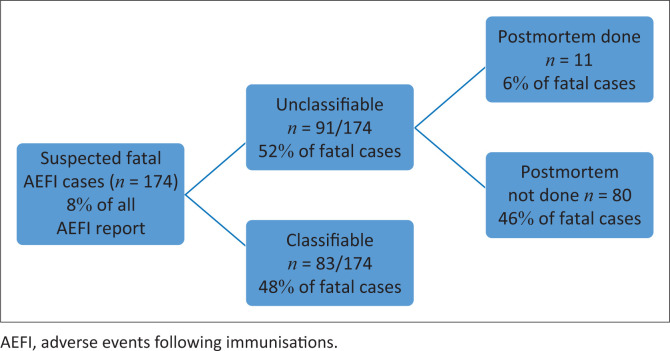
Suspected serious fatal adverse events following immunisations, postmortems and causality assessment.

Of the 174 reports of death as an AEFI, 83 out of 174 (48%) were classifiable and had sufficient information to allow an assessment of the causal relationship between the vaccine and cause of death (Figure 7).

However, 91 out of 174 (52%) were unclassifiable because of inadequate and missing information such as postmortem in 80 out of 174 (46%). Of all reported deaths, 158 out of 174 (91%) occurred after childhood vaccines and 16 out of 174 (9%) after an adult COVID-19 vaccine. All fatal AEFI cases were investigated; however, the limiting factor in establishing the cause of death was the lack of postmortem results because of the unavailability of postmortem facilities and, in some cases, the next of kin refused permission for a postmortem.

The National Causality Committee assessed 1104 AEFI reports initially using the Bradford Hill-based criteria from 1998 to 2013 and then the WHO causality assessment methodology (with revisions) from 2014 to 2019^23^ (Table 4).

**TABLE 4:** Causality assessment outcomes – Children vaccinees adverse events following immunisations and adult COVID-19 vaccinee adverse events following immunisations.

Causality assessment criteria	Children vaccines (*N* = 1767)				COVID-19 vaccines (*N* = 519)			
	Serious AEFI reports		Non-serious AEFI reports		Serious AEFI reports		Non-serious AEFI reports	
	*n*	%	*n*	%	*n*	%
1. Unclassifiable-inadequate information	103	5.8	28	1.6	18	3.5	5	1.0
2. Coincidental-underlying or emerging condition(s)	33	1.9	12	0.7	6	1.2	2	0.4
3. B2-Conflicting trends with causal association	0	0.0	1	0.1	1	0.2	0	0.0
4. B1-Temporal relationship, insufficient evidence	36	2.0	155	8.8	23	4.4	81	15.6
5. A4-Immunisation anxiety-related reaction	0	0.0	5	0.3	2	0.4	3	0.6
6. A3-Immunisation error-related reaction	16	0.9	94	5.3	0	0.0	1	0.2
7. A2-Vaccine quality defect-related reaction	0	0.0	0	0.0	0	0.0	0	0.0
8. A1-Vaccine product-related reaction	77	4.4	746	42.2	24	4.6	353	68.0
9. Unclassified/unconditional†	3	0.2	1	0.1	74	14.3	445	85.7
10. Unlikely†	90	5.1	4	0.2	N/A	-	N/A	-
11. Possible†	81	4.6	197	11.1	N/A	-	N/A	-

AEFI, adverse events following immunisation; COVID-19, coronavirus disease 2019; WHO, World Health Organization.

† , WHO Bradford Hill medicines-based causality assessment method before the WHO AEFI causality assessment algorithm method.

Seven hundred and forty-six (42.2%) non-serious AEFI reports were classified as vaccine product-related reactions (A1), 94 (5.3%) as immunisation error-related reactions (A3), 155 (8.8%) as demonstrating a temporal relationship but with insufficient evidence to prove a causal association (B1), 28 (1.6%) were unclassifiable because of inadequate information (D), 12 (0.7%) as coincidental underlying or emerging conditions (C) and 5 (0.3%) as immunisation anxiety-related reactions (A4). The serious AEFIs included 77 (4.4%) vaccine product-related reactions (A1), 16 (0.9%) immunisation error-related reactions (A3), 36 (2.0%) demonstrating a temporal relationship with insufficient evidence (B1), 103 (5.8%) unclassifiable because of inadequate information and 33 (1.9%) coincidental underlying or emerging conditions (C).

A total of 519 COVID-19 AEFIs were reported to the MCAZ NPC and ZEPI. Most adult COVID-19 vaccine AEFIs (*n* = 445/519; 85.7%) were non-serious, and many adverse events (87%) were resolved (Table 4). Causality assessment outcomes for non-serious AEFIs were 353 (68.0%) vaccine product-related reactions (A1), 81 (15.6%) demonstrating a temporal relationship with insufficient evidence (B1), 3 (0.6%) immunisation anxiety-related reactions (A4), 2 (0.4%) coincidental underlying or emerging conditions (C) and 1 (0.2%) immunisation error-related reaction (A3). Serious AEFI causality assessment outcomes were 24 (4.6%) A1 vaccine product-related reactions, 23 (4.4%) B1 temporal relationship with insufficient evidence, 6 (1.2%) coincidental underlying or emerging conditions, 2 (0.4%) A4 immunisation anxiety-related reactions and 18 (3.5%) unclassifiable because of inadequate information. Finally, outcomes for COVID-19 serious AEFI deaths were 10 (6.0%) unclassifiable because of inadequate information, 3 (1.7%) were coincidental underlying or emerging conditions and 3 (1.7%) were B1 temporal relationship because of insufficient evidence.

Immunisation error-related reactions were analysed, and the most common event was injection site abscess.

Injection site abscesses were most frequently reported after the pentavalent (DPT-HepB-Hib) vaccine (*n* = 52) followed by the Mumps-Rubella (MR) or Measles, Mumps and Rubella (MMR) vaccine (*n* = 17) and the BCG vaccine (*n* = 12).

The analysis focused mostly on those AEFIs where the reporter stated the component antigen(s) suspected of causing the injection site reaction. As these were mainly from combination vaccines, it was difficult to single out one component. For example, the pentavalent vaccine covers diphtheria, tetanus, pertussis, hepatitis B and *Haemophilus influenzae* type b (Hib).

A cluster of serious AEFIs was detected in 2009 with reports of nausea, vomiting and diarrhoea following measles vaccines during a campaign in Hurungwe District, Mashonaland West province. Following an investigation by the MCAZ NCP and ZEPI, the measles vaccine diluent batch samples did not meet sterility specifications, and the events were classified as immunisation error-related reactions. The outcome was MCAZ engagement with the vaccine procurement agent to strengthen vaccine storage and administration practices at vaccination clinics countrywide to prevent similar occurrences.

**FIGURE 8:**
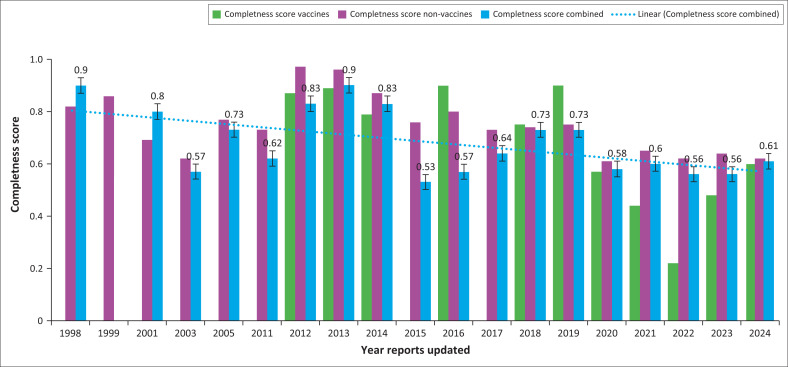
Zimbabwe VigiGrade completeness scores for individual case safety reports from 1998 to 2024.

The Zimbabwe VigiGrade completeness scores ranged from 0.40 to 0.90 for both vaccines and non-vaccines (medicines) (Figure 8).

In general, the scores for vaccines tended to be lower than for medications (vaccine range was 0.22–0.90 and medicines 0.61–0.97). However, the results demonstrated that Zimbabwe AEFI and adverse drug reaction (ADR) reports were of high quality, which unfortunately decreased after 2019 during the COVID-19 pandemic. The likely cause would have been limited health staff and resources, compounded by pressures such as infected staff, quarantines, lockdowns and staff attrition. Reports for 2022 were limited at the time of data analysis, showing only reports for quarters 1 and 2.

Medicines Control Authority of Zimbabwe underwent an assessment by the WHO using the GBT in May 2024, which demonstrated that 94% of the vigilance indicators were compliant, including AEFI surveillance. Medicines Control Authority of Zimbabwe successfully obtained the desired Maturity Level 3 (ML3), which indicates a stable, well-functioning system and includes vigilance, clinical trials regulation oversight, market control, market authorisation, regulatory inspection, licensing establishment, laboratory control and regulatory systems. Zimbabwe is the sixth African country to successfully achieve the desired overall ML3 of a well-functioning and stable NMRA.

## Discussion

We evaluated the Zimbabwean AEFI surveillance system over a 26-year period from 1998 to 2024. The surveillance system has not always been functional, but since 2009 AEFI reporting has improved markedly.

Over the 10-year period from 1998 to 2008, the surveillance system met the WHO minimum AEFI reporting rates of 10 per 100 000 surviving infants only in 2006. However, over the subsequent 15 years (from 2009 to 2024), the WHO benchmark was achieved in 12 out of 15 (80%) of the years.^13,24,25^ The highest AEFI reporting rates of 82 reports per 100 000 surviving infants occurred in 2024. The most frequently reported AEFIs were associated with those childhood vaccines administered as part of ZEPI or as part of the adult COVID-19 vaccine roll-out. The characteristics of the reported AEFIs, as classified by MedDRA (SOC and PT), were consistent with expected and common reactions (injection-site reactions, fever, rash). Of all the AEFI reports, 548 (24%) were classified as serious, consistent with the WHO recommendations to report all events (serious or non-serious) that are concerning to the vaccinee, parent or healthcare provider.

The improvement in AEFI reporting is likely to have been because of the joint MCAZ NPC and ZEPI enhanced AEFI surveillance training and the introduction of the VigiMobile application for AEFI reporting, which was launched in March 2023. All 11 provinces contributed AEFI reports, suggesting that the system functions throughout Zimbabwe. The VigiGrade completeness scores show an acceptable range, except for 2022, which was affected by the challenging conditions of the COVID-19 pandemic. The functionality of the AEFI surveillance system was demonstrated in 2009 with the detection of a cluster of immunisation errors during a measles vaccination campaign. The operation of the AEFI surveillance system is underpinned by a legislative framework as demonstrated by the GBT assessment; it met the desired international standard of a well-functioning national vigilance system at Maturity Level 3 in May 2023 and 2024, according to the WHO GBT independent assessment report.

Case investigation of serious AEFIs and causality assessment systems are well established in Zimbabwe. Of the 2286 reported AEFIs, 1828 (80%) underwent causality assessment review using the WHO causality assessment process and 458 (20%) were evaluated using the WHO–UMC causality categories. Causality assessment was conducted by the experienced National AEFI Committee, trained by the WHO over the years. In accordance with the WHO recommendations, the Committee consists of 10 specialists, including paediatricians, neonatologists, physicians, cardiologists, immunologists, epidemiologists, toxicologists, clinical pharmacologists and public health experts. In April 2017, the WHO coordinated an India–Zimbabwe inter-country study to assess the inter-rater reliability of the WHO AEFI causality assessment methodology, conducted by the National AEFI Committees of Zimbabwe and India. The study demonstrated a high degree of agreement between assessors using kappa coefficient analysis (personal communication, WHO). The qualitative aspect of the study results identified areas of the causality assessment that were subsequently improved using more accurate and clearer language, incorporated into the revised WHO AEFI causality assessment manual.^23^

Although we have demonstrated that Zimbabwe currently has a robust surveillance system for the detection, reporting, analysis and causality assessment of AEFIs, there are challenges and limitations. These include a lack of denominator data because there is no universal electronic vaccine register in Zimbabwe to determine the total number of exposed patients and no background reaction rates. A further limitation is that we were unable to stratify reporting rates of AEFIs for individual vaccines or vaccine combinations according to age or geographical location.

Reporting biases may arise because of media attention following serious AEFIs during vaccination campaigns. In the context of a pandemic vaccine, such as for COVID-19, the challenge is in addressing widespread misinformation disseminated via social media that focuses on vaccine safety and promotes vaccine hesitancy.^6,26^

There were 174 reports of death as an AEFI, of which 83 (48%) could be classified because the cause of death was determined and a valid diagnosis for the AEFIs was defined. A higher postmortem rate would have been required to reduce the rate of unclassifiable reports. However, we speculate that the postmortem rate in Zimbabwe is likely high for an LMIC. Some authors advocate that an autopsy should be mandatory in all deaths temporarily related to vaccine administration.^23^ It is also recommended that such postmortems should be conducted in line with the Letulle technique for clinical and forensic assessment in case of suspected death related to vaccines or minimally invasive autopsy tissue-sampling technique.^23,27^ Zimbabwe’s postmortem services have inadequate human resources for basic postmortem investigations.

Another limitation of the Zimbabwe surveillance system is the low rate of AEFI reporting in pregnancy, with no reports received during the period of review. However, the MCAZ (Mutare Hospital and Edith Opperman clinic, Harare) successfully participated in a WHO feasibility study of the Global Alignment of Immunization Safety Assessment in Pregnancy (GAIA) project case definitions based on levels of diagnostic certainty for pregnancy and neonatal outcomes. The study results showed that modification of the GAIA stillbirth definition could help avoid potential misclassification in LMICs.^14,28^ The study underscored the need for greater data literacy and inter-sectoral collaboration among healthcare providers, PV and health programme managers to promote harmonised approaches (case definitions and data elements) for capturing adverse outcomes of pregnancy.^14,15^

Access to timely and complete AEFI data is critical and could be facilitated using eHealth and mHealth.

We investigated mHealth active participant-centred surveillance using SMS as a potential AEFI surveillance tool in Zimbabwe (Zm-STARSS) from 2020 to 2021 in a randomised study where SMS gave a twofold increase in reporting compared with the passive control group and recommended its use for new pandemic vaccines.

Given the increasing penetration of mobile technology in Zimbabwe, it is possible to conduct such feasibility studies if more resources were available.^29^ Since April 2023, a new AEFI VigiMobile app linked to VigiFlow^®^ has been deployed countrywide for healthcare professionals, which has also resulted in increased reporting. Another study evaluating the impact of the AEFI VigiMobile application (app) is being conducted.

However, preliminary results showed that the quality of reporting is a challenge as the app does not have scope for AEFI case investigation for serious cases; hence, the manual AEFI case investigation form is still being used.

Given that causality assessment was not always conclusive for suspected AEFI fatalities because of inadequate postmortem information, there is a need to strengthen AEFI case investigations and expand postmortem facilities countrywide. The MCAZ NPC, in line with the WHO GBMT indicators for vigilance, should also conduct signal detection of AEFIs using the global AEFI database (VigiBase^®^) and disproportionate analysis. There should be a collaborative approach between government and academia, which could include the determination of background rates of adverse events of special interest.

## Conclusion

This study has demonstrated a functional AEFI surveillance system in Zimbabwe, which requires strengthening in the areas of timely AEFI detection and AEFI case investigation. Areas for improvement include completion of postmortems to enable causality assessment, VigiPoint^®^ disproportionate analysis signal detection and risk minimisation. Adverse events following immunisation case investigation initiatives ought to prioritise postmortems of fatal AEFI cases, as incomplete assessment of causation can severely compromise public confidence in vaccines. This requires adequate postmortem facilities at vaccination clinics and at referral district and provincial hospitals. Effective AEFI detection, case management, risk minimisation and promotion of vaccinee safety require ZEPI and MCAZ to use dependable, efficient and cost-effective electronic AEFI systems and to explore the use of VigiMobile and MAPC surveillance systems. Strong collaboration between the NIP and the NRA national pharmacovigilance centre is critical for strengthening the national AEFI surveillance system in a resource-limited country.

## References

[1] Sithole T, Mahlangu G, Salek S, Walker S. Evaluation of the regulatory review process in Zimbabwe: Challenges and opportunities. Ther Innov Regul Sci. 2021;55(3):474–489. 10.1007/s43441-020-00242-z 33387356 PMC8021537

[2] Clothier HJ, Hosking L, Crawford NW, et al. Bacillus Calmette-Guerin (BCG) vaccine adverse events in Victoria, Australia: Analysis of reports to an enhanced passive surveillance system. Drug Saf. 2015;38(1):79–86. 10.1007/s40264-014-0248-6 25475539

[3] Clothier HJ, Lawrie J, Russell MA, Kelly H, Buttery JP. Early signal detection of adverse events following influenza vaccination using proportional reporting ratio, Victoria, Australia. PLoS One. 2019;14(11):e0224702. 10.1371/journal.pone.0224702 31675362 PMC6824574

[4] Bahri P, Rägo L. CIOMS guide to vaccine safety communication–executive summary. Vaccine. 2019;37(3):401–408. 10.1016/j.vaccine.2018.11.082 30554796

[5] Akanmori BD, Traore T, Balakrishnan M, Maure C, Zuber P, Mihigo R. Vaccine safety and pharmacovigilance in the African region: Recent updates. J Immunol Sci. 2018. 10.29245/2578-3009/2018/si.1112

[6] Sampath V, Rabinowitz G, Shah M, et al. Vaccines and Allergic reactions: The past, the current COVID-19 pandemic, and future perspectives. Allergy. 2021;76(6):1640–1660. 10.1111/all.14840 33811364 PMC8251022

[7] Ampadu HH, Hoekman J, De Bruin ML, et al. Adverse drug reaction reporting in Africa and a comparison of individual case safety report characteristics between Africa and the rest of the world: Analyses of spontaneous reports in VigiBase^®^. Drug Saf. 2016;39(4):335–345. 10.1007/s40264-015-0387-4 26754924 PMC4796322

[8] Iessa N, Macolic Sarinic V, Ghazaryan L, et al. Smart Safety Surveillance (3S): Multi-country experience of implementing the 3S concepts and principles. Drug Safety. 2021;44(10):1085–1098. 10.1007/s40264-021-01100-z 34331675 PMC8325038

[9] Chandler RE. Optimizing safety surveillance for COVID-19 vaccines. Nat Rev Immunol. 2020;20(8):451–452. 10.1038/s41577-020-0372-8 32555401 PMC7298922

[10] Lei J, Balakrishnan MR, Gidudu JF, Zuber PL. Use of a new global indicator for vaccine safety surveillance and trends in adverse events following immunization reporting 2000–2015. Vaccine. 2018;36(12):1577–1582. 10.1016/j.vaccine.2018.02.012 29454518 PMC5857292

[11] Zvanaka S, Tsitsi J, Chonzi P, Shambira G, Gombe NT, Tshimanga M. Evaluation of the adverse events following immunizations surveillance system in Harare City, Zimbabwe, 2016: A descriptive cross sectional study. Pan Afr Med J. 2017;28(1):308. 10.11604/pamj.2017.28.308.12730 29721138 PMC5927576

[12] Ampadu HH, Esseku Y, Dodoo AN. Evidence-based pharmacovigilance for medicines used in public health programs in Africa. In: Evidence-Based Pharmacovigilance: Clinical and Quantitative Aspects. New York: Springer New York, 2018; p. 185–199.

[13] Shaum A, Mujuru HA, Takamiya M, et al. Enhanced surveillance for adverse events following immunization during the 2019 typhoid conjugate vaccine campaign in Harare, Zimbabwe. Vaccine. 2022;40(26):3573–3580. 10.1016/j.vaccine.2022.04.098 35568590 PMC10116805

[14] Sharan A, Stuurman AL, Jahagirdar S, et al. Estimating baseline rates of adverse perinatal and neonatal outcomes using a facility-based surveillance approach: A prospective observational study from the WHO Global Vaccine Safety Multi-Country Collaboration on safety in pregnancy. EClinicalMedicine. 2022;50:101506. 10.1016/j.eclinm.2022.101506 35770255 PMC9234094

[15] Sharan A, Jahagirdar S, Stuurman AL, et al. Operational lessons learned in conducting an international study on pharmacovigilance in pregnancy in resource-constrained settings: The WHO Global Vaccine safety Multi-Country collaboration project. Vaccine: X. 2022;11:100160. 10.1016/j.jvacx.2022.100160 35434599 PMC8993756

[16] Haider N, Hasan MN, Guitian J, et al. The disproportionate case–fatality ratio of COVID-19 between countries with the highest vaccination rates and the rest of the world. IJID Reg. 2023;6:159–166. 10.1016/j.ijregi.2023.01.011 36721772 PMC9881127

[17] Murewanhema G, Musuka G, Denhere K, Chingombe I, Mapingure MP, Dzinamarira T. The landscape of COVID-19 vaccination in Zimbabwe: A narrative review and analysis of the strengths, weaknesses, opportunities and threats of the programme. Vaccines. 2022;10(2):262. 10.3390/vaccines10020262 35214720 PMC8877028

[18] Tozzi AE, Asturias EJ, Balakrishnan MR, Halsey NA, Law B, Zuber PL. Assessment of causality of individual adverse events following immunization (AEFI): A WHO tool for global use. Vaccine. 2013;31(44):5041–5046. 10.1016/j.vaccine.2013.08.087 24021304

[19] Lee H, Kang H-Y, Cho S, et al. Causality assessment guidelines for adverse events following immunization with a focus on guillain–barré syndrome. Vaccines. 2020;8(1):101. 10.3390/vaccines8010101 32102455 PMC7157213

[20] Butler M, Tamborska A, Wood GK, et al. Considerations for causality assessment of neurological and neuropsychiatric complications of SARS-CoV-2 vaccines: From cerebral venous sinus thrombosis to functional neurological disorder. J Neurol Neurosurg Psychiatry. 2021;92(11):1144–1151. 10.1136/jnnp-2021-326924 34362855 PMC7614639

[21] Bergvall T, Norén GN, Lindquist M. VigiGrade: A tool to identify well-documented individual case reports and highlight systematic data quality issues. Drug Safety. 2014;37(1):65–77. 10.1007/s40264-013-0131-x 24343765 PMC6447519

[22] Guzman J, O’Connell E, Kikule K, Hafner T. The WHO Global Benchmarking Tool: A game changer for strengthening national regulatory capacity. BMJ Glob Health. 2020;5(8):e003181. 10.1136/bmjgh-2020-003181 PMC7418656 32784212

[23] Pomara C, Sessa F, Ciaccio M, et al. COVID-19 vaccine and death: Causality algorithm according to the WHO eligibility diagnosis. Diagnostics. 2021;11(6):955. 10.3390/diagnostics11060955 34073536 PMC8229116

[24] Gadzayi MR, Mukuzunga M, Chadambuka A, et al. An evaluation of the adverse events following immunization surveillance system: A case of the oral cholera vaccine mass campaign, Chimanimani and Chipinge Districts, Zimbabwe. 2019 [cited 2020 Dec 01]. Available from https://www.researchsquare.com/article/rs-64895/v1

[25] Masuka JT, Khoza S. Adverse events following immunisation (AEFI) reports from the Zimbabwe expanded programme on immunisation (ZEPI): An analysis of spontaneous reports in Vigibase^®^ from 1997 to 2017. BMC Public Health. 2019;19(1): 1166. 10.1186/s12889-019-7482-x 31455314 PMC6712865

[26] Mundagowa PT, Tozivepi SN, Chiyaka ET, Mukora-Mutseyekwa F, Makurumidze R. Assessment of COVID-19 vaccine hesitancy among Zimbabweans: A rapid national survey. PLoS One. 2022;17(4):e0266724. 10.1371/journal.pone.0266724 35446850 PMC9022878

[27] Chawana R, Baillie V, Izu A, et al. Potential of minimally invasive tissue sampling for attributing specific causes of childhood deaths in South Africa: A pilot, epidemiological study. Clin Infect Dis. 2019;69(Supplement_4):S361–S73. 10.1093/cid/ciz550 31598659 PMC6785686

[28] Stuurman AL, Sharan A, Jahagirdar S, et al. WHO global vaccine safety multi-country collaboration project on safety in pregnancy: Assessing the level of diagnostic certainty using standardized case definitions for perinatal and neonatal outcomes and maternal immunization. Vaccine: X. 2021;9:100123. 10.1016/j.jvacx.2021.100123 34825164 PMC8605263

[29] Psihogios A, Bota AB, Mithani SS, et al. A scoping review of active, participant-centred, digital adverse events following immunization (AEFI) surveillance: A Canadian immunization research network study. Vaccine. 2022;40(31):4065–4080. 10.1016/j.vaccine.2022.04.103 35680501

